# On the Impropriety of Mercurial Salivation

**Published:** 1842-03

**Authors:** Thomas Townsend

**Affiliations:** Wheeling, Virginia


### Art. II.—*On the Impropriety of Mercurial Salivation.* By Dr. Thomas Townsend, of Wheeling, Virginia.

A salivation from mercury, though not generally dangerous,is so great an inconvenience, and so much dreaded by patients, as frequently to deprive the physician of this valuable article of medicine. It is not at all surprising that patients dread a mercurial sore mouth, for in truth it is a loathsome, disgusting, and painful disease; and frequently worse than the disease for the cure of which it was produced. This being the case, as all candid medical men must acknowledge, the question presents itself, can mercury be used, and its agency as a remedy be obtained, without the inconvenience of a mercurial sore mouth? With the three exceptions of *acidity of the stomach, diseased* and *spongy gums*, and where the medicine cannot be made to *operate in due time*, I answer in the affirmative. If then, every medical man may obtain all the remedial agency and beneficial effects of mercury without its inconvenience; and if the very object of the profession is, not to inflict pain, but to relieve it, he is bound by every principle of uprightness, as well as humanity, not to add to the afflictions of his patients, the suffering and pain of a disgusting, loathsome, mercurial sore-mouth. If this be true, why salivate at all? what agency has salivation in the cure of disease? why then give mercurial preparations with a view to produce salivation? This is an important inquiry, and should receive the candid investigation of the medical profession. If ptyalism has not a decided physiological agency in the cure of disease, it certainly should not intentionally be produced.

All medicines act on the living system by stimulation. Every medicine has a specific action of its own, differing in some one or more particulars from the operation of every other medicine, on account of the peculiarity of its stimulation, or the particular part or branch of the system, upon which its impress is made. And this is as true of the preparations of mercury as of any other medicine. The most obvious specific effect of mercury is that of stimulating the salivary glands into such an increased action as either to amount to actual inflammation, or to a state of things bordering upon it; and attended with inflammation and ulceration about the mouths of their ducts. I will here make a quotation from the United States Dispensatory of Wood and Bache, for the purpose of showing what these gentlemen consider the modus operandi of mercury, and what no doubt is the generally received opinion of the medical professsion on that point, and more particularly what is considered *its constitutional effect.*

"Of the modus operandi of mercury, we can say nothing farther than that it seems to act through the medium of the circulation, and that it possesses a peculiar alterative power over the vital functions, which enables it in many cases to subvert diseased actions by substituting its own in their place. This alterative power is sometimes exerted, without being attended with any other vital phenomenon than the removal of the disease, while at other times it is attended with certain obvious effects, indicative of the agency of a potent stimulant. In the latter case, its operation is marked by a quickened circulation, with a frequent and jerking pulse; by an increase of activity imparted to the secretory functions, particularly those of the salivary glands and the liver; by an exaltation of the nervous sensibility; and, in short, a general excitement of the organic actions of the system. When its effects are not otherwise obvious than in the subversion of disease, its operation may be presumed to be the same as when it produces obvious stimulating effects, though so slight and imperceptible as altogether to escape notice.

"When mercury acts insensibly as an alterative, there is not the least disturbance of the circulation; but when it operates decidedly and obviously, it is very prone to let the brunt of its action fall upon the salivary glands, causing in many instances, an immoderate flow of saliva, and constituting the condition of things denominated ptyalism or salivation. Under these circumstances, to the alterative effects of the mineral, are added those of depletion and revulsion. Occasionally its depletory action is exhibited in an increased secretion of urine, or an immoderate flow of bile; and where ptyalism can not be induced, and either of these secretions becomes considerably augmented, the circumstance ought to be held equally conclusive of the constitutional effects as if the mouth had been affected."

I suppose from this quotation that the ptyalism is taken and held to be the obvious evidence of the constitutional effects of mercury. But during its administration, if an increased secretion of urine, or an immoderate flow of bile take place, we ought to hold this "equally conclusive of the constitutional effects as if the mouth had been affected." And "when its effects are no otherwise obvious than in the suppression of disease," and "its operation presumed to be the same," I

would ask why we should not "hold this equally conclusive of its constitutional effects as if the mouth had been affected." As it is so "very prone to let the brunt of its action fall upon the salivary glands," causing either "an immoderate flow of saliva," or an ulceration of the insides of the cheeks, and of the neighboring gums, and soreness of the teeth, &c., "constituting the condition of things denominated ptyalism or salivation," may we not with great propriety call this the *local specific effect* of mercury? In other words, does not salivation furnish the obvious evidence that the medicine has produced its local specific effect?

The specific effect of this article is salivation. If mercury be determined to the mouth it will expend its whole force about the mouth and salivary glands. Every practitioner of much experience knows, that if he have a patient with a mercurial sore mouth, and in this condition of things he give a dose of calomel as a cathartic, it will not operate, but will go to the mouth and make it sorer; and he will be compelled to prescribe some other cathartic. The ptyalism is so great an inconvenience to the patient that, if possible, the remedial agency of mercury should, at all times, be obtained by what Wood and Bache call its "alterative power," which is more properly "its constitutional effects" than ptyalism, the action of which is, at first, entirely local and confined to the vessels about the mouth. But when the inflammation of the mouths of the salivary ducts, and of the gums, and the periosteum of the fangs of the teeth, becomes considerable, the irritation from this local inflammation produces sympathetic fever, for the same reason, and on the same physiological principle as the local irritation and pain from the inflammation of wounds, such, for instance, as amputation, or from other local inflammations followed by sympathetic fever. This inflammation and sympathetic fever, although attended by "a quickened circulation and with a jerking pulse," are not sufficient, in my estimation, to establish the opinion that this is "the constitutional effect" of mercury, but rather its specific effect. If the term "constitutional effect" be applicable at all to the effects of mercury in its modus operandi, it should rather be applied to the effects which it produces in other parts or branches of the system, than the salivary glands. Thus it will be seen that, instead of adopting the commonly received opinion, that a mercurial salivation is the evidence of the constitutional effects of the mineral, I consider this circumstance the evidence, that it has ceased to be a general or constitutional remedy, and has become a local irritant. For so soon as the medicine begins to be determined to the mouth, all that may be given will go the same way, and keep the mouth sore so long as the medicine may be continued. If we wish the agency and remedial power of this medicine to operate on other portions of the system, the local or specific effect should be avoided. The question then very naturally occurs, can this be done? I answer it can.

In the early part of my practice I entertained the common opinion, that salivation ought to be frequently produced, not only in syphilis and fevers, but in chronic diseases. In two cases of fever occurring nearly at the same time, I prescribed calomel, and directed the patients to drink as much cold water as they wished, and told them that if the mouth became sore so much the better. I visited them daily, and they both recovered without being salivated. These cases led me to make observations for myself, the result of which was, that cold water for common drink, did not, and would not, determine calomel to the mouth, and make it salivate, according to the generally received opinion. At a later period, from having so frequently observed a salivation take place when acid drinks, such as tamarind water, lemonade, or cream of tartar, were used during the administration of calomel, I came to the conclusion that acids must have something to do with the production of this effect, I then began to experiment, until I became fully satisfied, that the use of acid drinks during the administration of calomel, and the salivation, stood to each other in the relation of cause and effect. After becoming satisfied of this fact, I forbade acids altogether, during the use of calomel; and since that time I have had no case of salivation, when patients would rigidly follow directions. After a few years' observation and experience of this mode of using and managing mercury, I became fully satisfied of the superiority of obtaining its remedial agency and alterative effects without the troublesome inconvenience of a salivation.

I have no hesitation in saying that a cure is more speedily and better performed by calomel, or any other mercurial preparation, without ptyalism than with it. As mercury operates through the medium of the sanguiferous system, there is no doubt in my mind that it operates by stimulating into increased action both the secernent and absorbent vessels of the system. By a judicious combination with other articles, which operate on the secernent or absorbent system, mercury may be determined to the one or the other, and in this way act as an alterative, so as to change diseased action, and restore the system to a healthy condition, much better than when permitted to go to the mouth and produce salivation. Colchicum and sanguinaria, in certain doses, act as alteratives, and change diseased action, without any material evacuation, either by vomiting, purging, or sweating; and such, I apprehend, is the operation of mercury when, as is said in the Dispensatory, "it subverts diseased action without any obvious effect" on the circulation. But it is usually followed by evacuations from the intestinal canal. To this effect of operating on the alimentary canal, after having made its peculiar impression on other parts of the system to which it had been directed, may be attributed the superiority of mercury as an alterative over almost every other article in the materia medica. In fact, if it be prevented from being determined to the mouth, it may be made to operate on the capillary arteries, veins, and absorbents, and the whole glandular system.

I consider its operation on the salivary glands as definitely specific as that of the specific operation of secale cornutum on the muscular fibres of the uterus. If permitted to go to the salivary glands, it will expend the whole force of its stimulating power upon those parts as certainly as the latter medicine exerts its action upon the uterus. And, as the associated action of the abdominal and other muscles results from the increased activity of those of the uterus, so the increased activity of the vessels about the mouth may bring into association, through the medium of nervous influence, an increased activity of the whole sanguiferous system, as manifested by the "frequent jerking pulse."

During the last twelve or fourteen years, I have had no case of salivation in my practice, except such as may be considered accidental—that is, where patients, contrary to directions, while taking mercury have used acids, or where the stomach was loaded with acid at the commencement of using it, or where the medicine could not be made to operate so as to be carried out of the system in due time, or where the gums were diseased, spongy, and inflamed, and the action already determined to the mouth. The first of these accidental circumstances depends entirely on the patient and his nurses. The second may, in general, be easily remedied by giving before the administration of calomel, a sufficient quantity of bicarbonate of soda to correct the existing acid, and by giving a little from time to time to neutralise that which may continue to be generated, until the medicine shall have operated and been carried out of the system. I know no remedy for the diseased and spongy gums, but to go to a dentist, and get the gums cured and the mouth put in order before the individual becomes otherwise sick! If the patient has neglected this, when he falls sick, and mercury becomes necessary, he must run the risk of a sore mouth, and submit, when it takes place, with the best grace he can. With regard to the slow and constipated state of the bowels, which retains the article too long in the system, or until it becomes determined to the mouth—when we have reason to suspect sore mouth, this can only be prevented by using other cathartics more speedy in their operation, and assisting them by enemata. I know no other circumstances, when acids are strictly avoided, that will determine calomel to the mouth, and make it salivate, but the three just enumerated.

I adopted the practice of so administering and managing calomel as to avoid salivation, under the full conviction that its action when determined to the glands about the mouth, is local, and not general over the constitution—that when it produces ptyalism, it acts as a local irritant, and that if we wish "its constitutional effects," or rather its operation on other parts of the system than the salivary glands, it must be so managed as to prevent its determination to the mouth.

The view I entertain, that salivation is not the constitutional, but the local specific effect of mercury, has been greatly strengthened from finding every medical man, with whom I have conversed on this subject, and who advocated the old doctrine, unable to give a rational physiological reason why a patient laboring under bilious remittent fever, for instance, should be salivated; or why salivation should be resorted to in a case of enlargement or chronic inflammation of the liver or spleen; or why this effect should be produced for the cure of incipient cataract, or removal of opacity of the cornea from abscess upon that membrane. Can any one explain the modus operandi, so to speak, of salivation, in the cure of any one of these diseases—unless by referring it to the doctrine of sympathy?—a term which, Dr. Porter says, "will cloak ignorance under the appearance of science, as well as any other," and cut off all farther inquiry and investigation, so as to be passed off as an explanation of what is not understood. I can not believe, then, that ptyalism is the constitutional effect of mercury, until a clear, rational, and physiological explanation can be given why the local inflammation about the mouth is constitutional. Until this can be done, I must be permitted to consider this local inflammation as the local specific effect; and that it is only in the absence, or rather avoidance of this local effect, that we are able to avail ourselves in full of the general remedial agency of this medicine in other parts in which we wish it to operate, or to which we wish its agency directed.

I hope the reader will not hastily condemn these views because they may be new to him, but will try them by the test of experience, and if he finds them to be true, have candor and magnanimity enough to acknowledge their correctness. But if on a full and fair trial by experiment he finds them untrue, then I shall expect him to give a rational physiological explanation of the modus operandi of mercury, such as will show conclusively their fallacy. I have been long intending to publish these views, but felt fearful, because they may not be considered orthodox. But when I reflect that the profession to which I belong cannot be considered as having arrived at that state of perfection, at which investigation should cease, because the profession is incapable of farther improvement; and when I reflect further, that a physician who makes no attempt to improve his profession, lives to but little purpose, I have at last ventured to submit these views, with the hope that if they do nothing more, they will induce those whose talents, acquirements, and station so much better fit them for the purpose, to test by experience my modified method of managing and using this potent, useful, and necessary article of the materia medica—an article indeed, for which, in many cases, we have no substitute. And besides this, and as a further reason for desiring others to try this method of employing mercury, I may remark, that one of the professed objects of the medical profession is, to relieve the distress, and pain, and suffering of our fellow beings. This being the case, humanity requires, not only that we should do this to the utmost of our ability, but that in doing it, we should not give any additional pain, or by the use of our remedial agents, produce any increase of suffering that can be avoided.

As I feel assured that all the beneficial agency may be had from the operation of mercury, as an alterative or cathartic, without the inconvenience of a mercurial sore mouth; and, as I believe, further, that the general agency and efficacy are more certain and more efficient, more agreeable to the patient, and more consistent with the principles of humanity, I hope to live to see the day when no enlightened, high-minded and honorable physician will designedly produce a mercurial salivation; but will unite, as he ought to do, in removing any prejudice in the minds of the people, not only against this drug, but against the medical profession for its employment.

Jan. 13, 1842.

